# Use of 3D Organoids as a Model to Study Idiopathic Form of Parkinson’s Disease

**DOI:** 10.3390/ijms21030694

**Published:** 2020-01-21

**Authors:** Paula Chlebanowska, Anna Tejchman, Maciej Sułkowski, Klaudia Skrzypek, Marcin Majka

**Affiliations:** Jagiellonian University Medical College, Faculty of Medicine, Institute of Pediatrics, Department of Transplantation, Wielicka 265, 30-663 Kraków, Poland; paulalota0@gmail.com (P.C.); tejchman.anna@gmail.com (A.T.); maciej.sulkowski@gmail.com (M.S.); klaudia.skrzypek@uj.edu.pl (K.S.)

**Keywords:** organoid, Parkinson’s disease, iPS cells, 3D model, idiopathic, LMX1A, TH, FOXA2, PTX3

## Abstract

Organoids are becoming particularly popular in modeling diseases that are difficult to reproduce in animals, due to anatomical differences in the structure of a given organ. Thus, they are a bridge between the in vitro and in vivo models. Human midbrain is one of the structures that is currently being intensively reproduced in organoids for modeling Parkinson’s disease (PD). Thanks to three-dimensional (3D) architecture and the use of induced pluripotent stem cells (iPSCs) differentiation into organoids, it has been possible to recapitulate a complicated network of dopaminergic neurons. In this work, we present the first organoid model for an idiopathic form of PD. iPSCs were generated from peripheral blood mononuclear cells of healthy volunteers and patients with the idiopathic form of PD by transduction with Sendai viral vector. iPSCs were differentiated into a large multicellular organoid-like structure. The mature organoids displayed expression of neuronal early and late markers. Interestingly, we observed statistical differences in the expression levels of LIM homeobox transcription factor alpha (early) and tyrosine hydroxylase (late) markers between organoids from PD patient and healthy volunteer. The obtained results show immense potential for the application of 3D human organoids in studying the neurodegenerative disease and modeling cellular interactions within the human brain.

## 1. Introduction

The generation of the induced pluripotent stem cells (iPSCs) by Yamanaka’s group was a milestone for regenerative medicine. Yamanaka discovered that factors that are responsible for the parental state of embryonic stem cells (ES) might also confer pluripotency in somatic cells. 24 genes that are responsible for pluripotency in early embryos and ES cells were selected as candidates. Among them, four factors were discovered to be necessary and sufficient for the transformation of somatic cells into iPSCs in a process, called reprogramming. Those factors are Sox2, Oct3/4, Klf4, and c-Myc [1,2].

Reprogramming technology has opened new possibilities in the field of cellular replacement, regenerative therapy, and disease modeling. iPSCs can be generated from somatic cells, which allows for studies using patients derived cells. Moreover, the generation of iPSCs enables the development of in vitro models used for personalized drug screening [3,4]. Many patient-derived iPSCs cell lines were obtained and served as models of different diseases, such as, for example diabetes type I, LEOPARD syndrome, and others [5,6,7]. Interestingly, iPSCs were also used in studies on brain pathologies to investigate the mechanisms of neurodevelopmental and neurodegenerative diseases that are linked to aging. For example, iPSCs turned out to be good models for Huntington’ disease [5], Parkinson’s disease (PD) [7], Down’s syndrome [5], Alzheimer’s disease [8,9], autosomal dominant lateral temporal epilepsy [10], Creutzfeldt–Jakob disease [11] and neuronal ceroid lipofuscinosis [12]. iPSCs also opened up the possibility of studying diseases with unknown pathogenesis. Another advantage of iPSCs is the elimination of ethical concerns regarding embryonic stem cells (ESCs) utilization.

The introduction of iPSCs has enabled more accurate modeling of diseases in vitro. However, attempts are still being made to improve the differentiation of these cells. The missing link between two-dimensional (2D) cultures and in vivo animal disease models are three-dimensional (3D) cultures such as organoids [13,14,15,16]. The development of a 3D model of differentiation is based on the ability of iPSCs to self-organize. Organoids create a favorable micro-environmental niche for differentiation and maturation of cells due to their structure [17].

Recent advances have enabled the generation of specific brain organoids. They represent the organized structures that are formed by progenitors, neurons, and glial cells, which resemble the architecture of human brain. Furthermore, organoids may be composed of cells that are specific for particular brain regions, such as midbrain or forebrain [15,17]. These organoids can be generated from iPSCs that are derived from patients, which creates an opportunity for disease modeling, studies on the development and progression of disease, as well as for drug testing. Several studies on brain pathologies have already involved iPSCs derived organoids [8,11,12]. Thus, the 3D model of PD is an excellent tool for studying the pathomechanisms of this disease [18].

The aim of this study was to characterize iPSCs that were obtained from healthy volunteers and PD patients with idiopathic form of the disease using the 3D model. Midbrain organoids were generated from iPSCs cells derived from peripheral blood mononuclear cells (PBMCs). The pluripotency of iPSCs was analyzed using techniques for the evaluation of pluripotency markers, alkaline phosphatase expression, and abilities to form teratomas in immunodeficient mice. After positive characterization, the iPSCs were differentiated into dopaminergic neurons (mDA) in the midbrain organoid model. Differentiation was evaluated by the analysis of markers characteristic for mDA at various stages of development. We aimed to verify whether iPSCs from idiopathic PD patients have distinct differentiation potential during midbrain organoids formation.

## 2. Results

### 2.1. Generation of Induced Pluripotent Stem Cells from Peripheral Blood Mononuclear Cells

**Figure 1:**
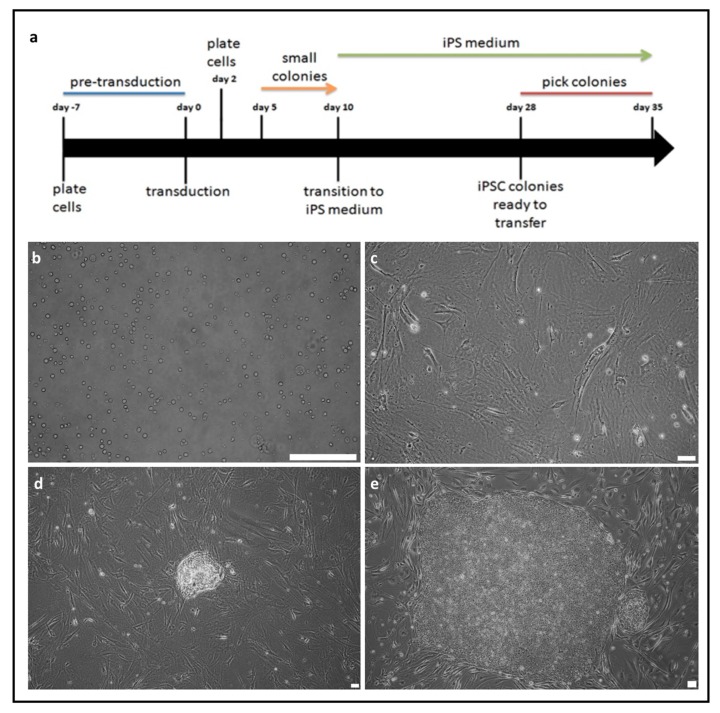
Scheme of induced pluripotent stem cells (iPSCs) generation from peripheral blood mononuclear cells (PBMCs) (**a**). (**b**) On day 0 PBMCs cells are transduced with Sendai viral vector. (**c**) On day 2 the cells are transferred onto plate with iMEFs. (**d**) On day 10 formation of the first colonies is observed. (**e**) On day 30 colonies are ready to be transferred. White scale bar: (**b** and **d**) 100 μm (**c** and **e**) 50 μm. Representative images of iPSCs generation from four different donors are shown.

PBMCs were obtained from the blood of two healthy volunteers and two PD patients (Figure 1a). They were reprogrammed using the non-integrating Sendai viral vector. Differences in morphology of the cells were visible during reprogramming. PBMCs that were cultured in the expansion medium displayed rounded shape (Figure 1b), whereas, two days after transduction, the morphology of infected cells was not modified (Figure 1c). Small colonies were noticeable between day 5 and 10 (Figure 1d). When iPSCs colonies were ready to transfer, their edges were regular and small cells were tightly packed (Figure 1e). Based on their morphology, the best colonies were selected. The acquired iPSCs clones were expanded up to 10th passage. Figure 1 shows representative images of iPSCs generation from four different donors.

### 2.2. Characterization of Induced Pluripotent Stem Cells Generated from Healthy Volunteers and Parkinson’s Disease Patients

**Figure 2:**
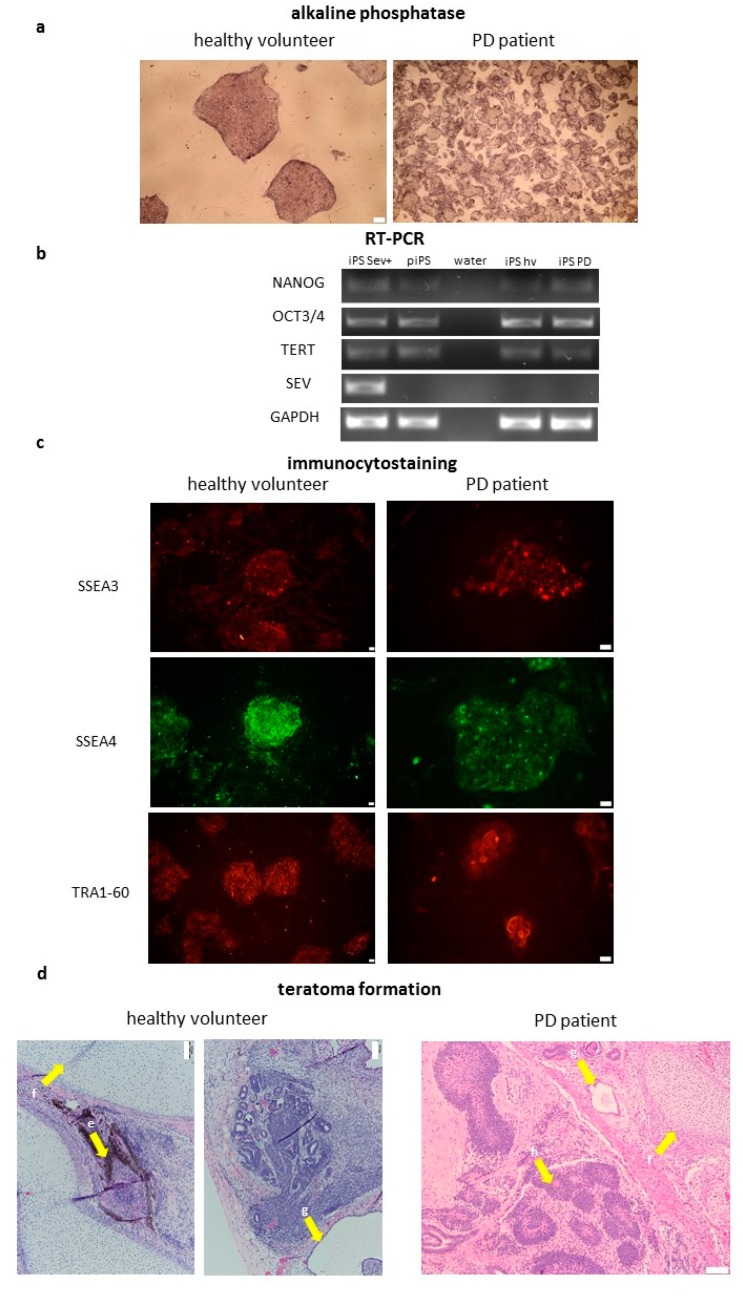
Characterization of PMBCs-derived iPSCs lines from two healthy volunteers and two Parkinson’s disease (PD) patients. Representative images from several iPSCs clones derived from four donors are shown. (**a**) iPSCs lines are positive for alkaline phosphatase. (**b**) iPSCs lines (iPS Sev+-iPS with Sendai viral vector at early passage; piPS—protein-induced iPS; iPS hv—iPS healthy volunteer; iPS PD—iPS Parkinson Disease) express endogenous pluripotency genes: NANOG, OCT3/4, TERT; viral transgene (SeV) is silenced after 10th passage. Water was used as a negative control. (**c**) iPSCs lines express surface markers SSEA3, SSEA4, and TRA1-60. (**d**) iPSCs lines form teratomas in vivo. (e) pigmented cells, (f) cartilage, (g) secretory epithelium, (h) neuroectodermal rosettes. NANOG—homebox protein NANOG; OCT3/4—octamer binding transcription factor 3/4; TERT—telomerase; GAPDH—housekeeping gene, glyceraldehyde 3-phosphate dehydrogenase; SEV—primer specific for Sendai Virus genome; SSEA3—stage specific embryonic antigen 3; SSEA4—stage specific embryonic antigen 4; TRA1-60—podocalyxin. All of the images (**a**–**d**): white scale bar, 100 μm.

After establishing iPSCs lines, they were characterized according to current standards [1,19]. The generated cell lines expressed alkaline phosphatase (Figure 2a) and endogenous pluripotency markers, such as NANOG, OCT3, and telomerase (TERT) at the mRNA level (Figure 2b). Commercially available protein-induced iPSCs line (piPS) was used as a positive control. iPSCs expressed also surface markers, such as SSEA3, SSEA4, and TRA1-60 (Figure 2c). They were able to generate teratomas in vivo. Histopathological analysis of the formed tumors revealed the presence of all three germ layers (Figure 2d). The presence of the Sendai viral vector has been checked. At the 10th passage, the cell lines were free from the Sendai viral vector, which suggested that viral transgen (Sev) was silenced (Figure 2b). These results suggest efficient reprogramming of PBMC from healthy volunteers and PD patients. No significant differences were observed in the generated cell lines.

### 2.3. Organoids Formation from Induced Pluripotent Stem Cells from Healthy Volunteer and Parkinson’s Disease Patient

**Figure 3:**
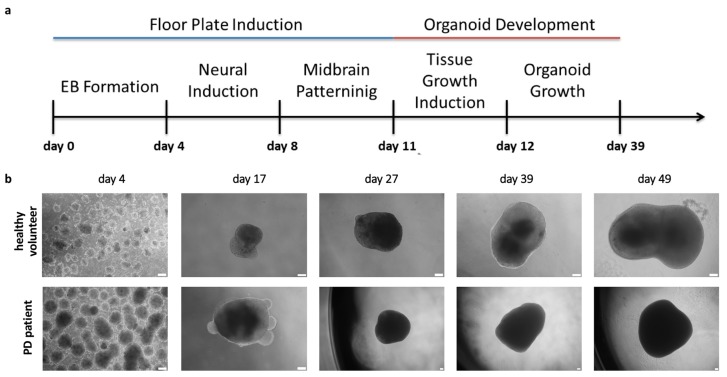
Scheme of organoids generation and morphology of organoids from healthy volunteer and PD patient. (**a**) Diagrams presenting strategy of generation of midbrain organoids. (**b**) Morphology of organoids structures at different time points at day 4, 17, 27, 39, and 49. The pictures show representative images from six different organoids per donor in two independent experiments (three organoids in each experiment). In each experiment organoids were generated from 48 embryoid bodies. White scale bar, 200 μm.

iPSCs were differentiated into organoids using the protocol that was described by Jo. et al. 2016 (Figure 3a) [15]. 48 embryoid bodies per patient were used to generate organoids from the selected iPSCs clone from healthy volunteer and PD patient and the experiment was repeated two times. Organoids (*n* = 3 in each experiment) were collected on day 4th, 17th, 27th, 39th, and 49th for analysis (Figure 3b). After day 49th, we observed reduced organoid growth. As observed in Figure 3 (representative images), the morphology of organoids was similar and no significant differences were detected between the groups.

**Figure 4:**
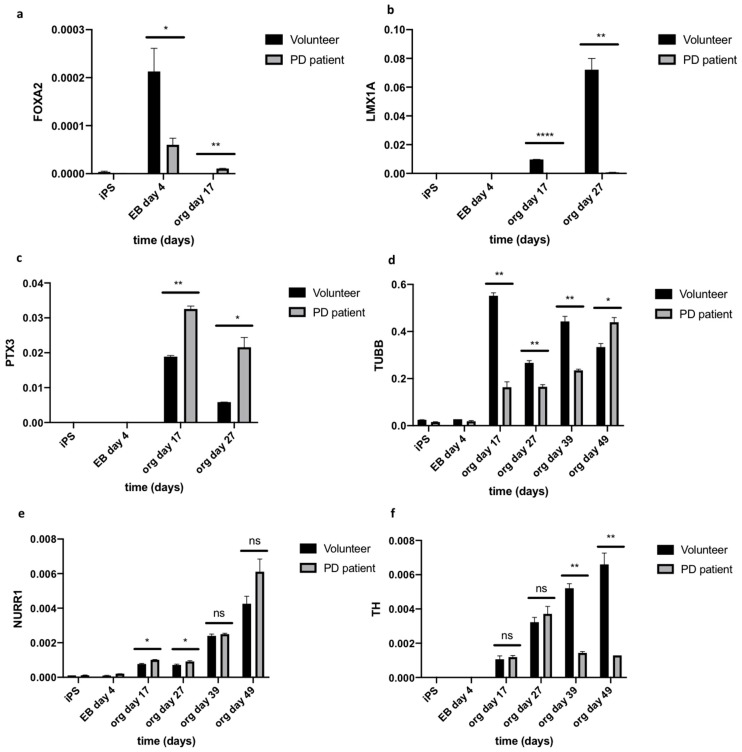
Expression (RT-qPCR) of early (FOXA2, LMX1A, PTX3) and late neurons markers (TUBB3, NURR1, TH) in organoids derived from healthy volunteer and PD patient. At each time point (day 0, 4, 17, 27, 39, 49 of differentiation) the RNA samples are collected from three organoids (*n* = 3). (**a**) The expression of FOXA2 is significantly lower in PD organoids versus control organoids on day 4, whereas it is higher comparing to control on day 17. * *p* < 0.05, ** *p* < 0.01 (**b**) The expression of LMX1A is significantly higher in control organoids versus PD organoids on day 17 and 27. **** *p* < 0.0001, ** *p* < 0.01. (**c**) The expression of PTX3 is significantly higher in PD organoids versus control on day 17 and 27. ** *p* < 0,01, * *p* < 0.05. (**d**) The expression of TUBB3 is higher in control organoids on day 17, 27, 39. The expression of TUBB3 is higher in PD organoids versus control organoids on day 49. Day 17 ** *p* < 0.01, day 27 ** *p* < 0.01, day 39 ** *p* < 0.01, day 49 * *p* < 0.05. (**e**) NURR1 expression shows differences between groups on day 17 and 27. Day 17 * *p* < 0.05, day 27 * *p* < 0.05. (**f**) The expression of tyrosine hydroxylase (TH) is higher in control organoids at each time point from day 17. The expression of TH in PD organoids is stable on day 27 and is lower on day 39 and 49. Day 39 ** *p* < 0.01, day 49 ** *p* < 0.01. TUBB3—beta-III-tubulin, TH—tyrosine hydroxylase, NURR1—nuclear receptor related 1 protein, LMX1A—LIM homeobox transcription factor 1 alpha, FOXA2—forkhead box A2, PTX3—pentraxin-related protein. Relative expression of all genes to GAPDH were validated by RT-qPCR. Student t-test was performed to verify statistical relevant between the compared groups in each time point. Three independent organoids were analyzed per group (*n* = 3). Those organoids were generated from 48 embryoid bodies per donor (healthy volunteer and PD patient) and the experiments were repeated two times. The graphs show representative results from three different organoids. The data represent the mean ± SD.

The expression of early (FOXA2, LMX1A, PTX3) and late (NURR1, TH, TUBB) differentiation markers was analyzed on days 0, 4, 17, 27, 39, and 49. Significant differences in gene expression were detected between healthy volunteer and Parkinson’s disease groups. The expression of FOXA2 (forkhead box A2) was detected on day 4, but the level of its expression was statistically higher (3.55 fold) in organoids derived from healthy volunteer, and then from PD patient’s organoids (Figure 4a). Significantly increased expression of LIM homeobox transcription factor alpha (LMX1A) on day 17 (102.55 fold) and 27 (104.08 fold) was also noted in organoids from healthy volunteers as compared to PD patients’ organoids (Figure 4b). The organoids from both groups showed the expression of PTX3 (pentraxin-related protein) at day 17, and then the expression decreased. PTX3 expression was higher on days 17 (1.72 fold) and 27 (3.69 fold) in organoids from PD patient than healthy volunteer (Figure 4c).

NURR1 (nuclear receptor related 1 protein) is expressed in post-mitotic mDA progenitors [20]. We found that, from day 17 to 49, the level of NURR1 was raising in both groups. However, we did not observe significant differences in the expression of NURR1 between organoids from healthy volunteers and PD patients (Figure 4e). Interestingly, in contrast to NURR1, the level of tyrosine hydroxylase (TH) increased in healthy volunteer organoids over time, whereas, in PD patients’, organoids tyrosine hydroxylase (TH) expression reached the plateau at day 27. TH expression level was significantly lower on day 39 (3.62 fold) and 49 (5.13 fold) in PD organoids when compared to healthy volunteer’s organoids. It suggests that the level of TH in PD organoids was increasing until day 27 and significantly decreased on days 39 and 49 (Figure 4f). Furthermore, we observed that level of beta-III-tubulin (TUBB) was lower on day 17 (3.38 fold), 27 (1.61 fold), and 39 (1.89 fold) in PD patient’s organoids as compared to healthy volunteer’s organoids. However, the situation changed on day 49, when the expression of TUBB was approximately at the same level (0.76 fold) (Figure 4d).

**Figure 5:**
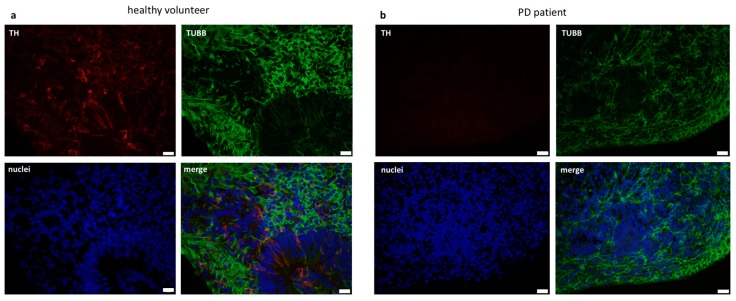
Differences in TH protein level in organoids from healthy volunteer and PD patient. The representative images show immunocytofluorescent staining for TUBB3 and TH in organoids (**a**) TUBB3 and TH is expressed in control organoids on day 39. White scale bar, 20 μm. (**b**) PD organoids express TUBB3 and do not express TH on day 39. White scale bar, 20 μm. TUBB3—beta-III-tubulin (green), TH—tyrosine hydroxylase (red), Hoechst (blue).

We evaluated TUBB and TH expression on day 39 while using immunostaining to confirm this data at protein level. TH collocated with TUBB in the cells. Interestingly, TUBB positive neurons were also positive for TH, but only in organoids from healthy volunteers (Figure 5). Those results showed significant differences in the expression of neuronal differentiation markers between the healthy volunteer and Parkinson’s disease groups.

These data might suggest that, in the idiopathic form of Parkinson’s disease, there is a defect in neuronal development process manifesting itself in 3D midbrain organoids.

## 3. Discussion

In recent years, progress has been made in the field of disease modeling. This has been achieved thanks to the 3D organoids technology, which, through their three-dimensional structure, allows for the reconstruction of tissue architecture [15]. Moreover, the possibility of receiving iPSCs from a particular person enables a personalized study on the mechanisms that take place during progression of the disease [18,21,22].

The literature offers various protocols for obtaining mDA, however, most of them do not consider the midbrain structure. The solution to this problem is offered by the organoid creation technique; however, only genetic form of PD with LRRK2-G2019S mutation has been studied while using the 3D organoid model so far [18]. In this study, we present, for the first time, a model of midbrain organoids that is based on iPSCs obtained from PD patient with an idiopathic form of the disease.

LMX1A and FOXA2 positive midbrain floor plate progenitors were obtained as a result of the application of the above protocol [21]. LMX1A plays a key role during the development of midbrain dopaminergic progenitors [23]. LMX1A/B regulate functions of mitochondria and survival of adult mDA neurons [24]. The human polymorphism of LMX1A/B has been associated with PD [25]. Previous publications have shown that FOXA2 is required for DA neuron development in vivo [26]. Neurons are protected against toxins as a result of the interaction between FOXA2 and NURR1, but these transcription factors are lost during degenerative processes and aging [27]. Interestingly, in our study we observed significantly higher level of midbrain floor plate progenitors, such as FOXA2 and LMX1A, during the differentiation processes in healthy volunteer’s organoids, in comparison to PD organoids.

PTX3 is another important factor that is involved in the development and survival of mDA neurons [28]. In our model of midbrain organoids derived from PD iPSCs, the level of *PTX3* gene expression was higher when compared with the control organoids. Similar results have been described before [29]. The level of *PTX3* was increased in PD patients and the literature showed significant correlation between a higher level of *PTX3* with disease severity in PD patients [30].

NURR1 has been shown to be responsible for the differentiation of early mDA progenitors into mature TH positive neurons [31]. However, NURR1 deficiency is associated with induction of mDA neuron dysfunction in PD, in addition to its role in normal brain development [32]. In our study, no differences in the expression level of *NURR1* gene in organoids derived from iPSCs from PD patient were observed when compared to control. Despite this, a decrease in *TH* gene expression was observed on day 39 and 49 in PD patient’s derived organoids compared to the control. Moreover, we demonstrated very low protein level of TH in the PD derived organoids in comparison to TH protein level in healthy volunteer’s derived control organoids. The low TH expression in organoids from PD patient is associated with the phenomenon of dopaminergic neuron degeneration in these patients [33].

Thus, our results demonstrated, for the first time, that the idiopathic background of PD patients could influence the generation of 3D midbrain organoids. Similar results have been shown in the previous research of Smits et. al., 2019, where the influence of genetic background of PD on organoid development has been described [18]. Our novel model identified several crucial gene candidates that might be responsible for development of idiopathic form of PD. Among those candidates are the following neuronal markers: TH, PTX3, LMX1A, and FOXA2. The dysregulation of their expression might lead to higher vulnerability of neurons to damage and degeneration. To conclude, the organoids formation is powerful tool for in vitro modeling of PD disease. The patient specific molecular characteristic of PD disease can be replicated in 3D model and help to study the pathology of the disease in vitro.

## 4. Materials and Methods

### 4.1. Cell Culture

iPSCs were generated from peripheral blood mononuclear cells (PBMCs) that were collected from four different donors while using Sendai viral vector-based reprogramming or obtained commercially (piPS, SBI System Biosciences). They were cultured in serum-free iPSCs medium containing DMEM/F12 supplemented with 20% KSR, 2 mM Glutamax, 100 U/mL Penicillin/Streptomycin, 100 µM Non-Essential Amino Acids, 10 ng/mL bFGF (all from Thermo Fisher Scientific, Waltham, MA, USA) and 100 µM β-mercaptoethanol (Sigma–Aldrich, Saint Louis, MO, USA) on dishes that were coated with gelatin (Sigma–Aldrich) with a feeder layer of the mouse embryonic fibroblasts (MEFs) inactivated with mitomycin C (Sigma–Aldrich). The medium was changed every day. The iPSCs were routinely passaged with accutase (Lonza, Basel, Switzerland) and seeded on new dishes with density 1:4 - 1:10 in medium with 10 µM ROCK inhibitor Y-27632 (Sigma–Aldrich). iPSCs were cryopreserved in liquid nitrogen in freezing medium consisting of 90% fetal bovine serum (FBS) (Eurx, Gdansk, Poland) and 10% DMSO (Sigma–Aldrich). One hour before freezing cells were incubated with 10µM ROCK inhibitor. For the formation of midbrain organoid, the cells were cultured in feeder free conditions, on dishes that were coated with growth factor-reduced Matrigel (Corning, New York, NY, USA).

PBMCs were cultured in Expansion Medium containing serum free medium QBSF-60 (VWR, Radnor, PA, USA), 100 U/mL Penicillin/Streptomycin, 10 µL/mL ascorbic acid, and growth factors: 50 ng/mL SCF, 10 ng/mL IL-3, 2 U/mL EPO, 40 ng/mL IGF-1, 1,5 µM dexamethasone (all from Peprotech, London, United Kingdom). MEFs were cultured in Dulbecco’s Modified Eagle Medium (DMEM) with 4.5 g/L glucose supplemented (Thermo Fisher Scientific) with 10% FBS, 2 mM l-glutamine (Thermo Fisher Scientific), and 100 U/mL Penicillin/Streptomycin. The cells were cultured in a humidified atmosphere of 5% CO_2_ at 37 °C.

### 4.2. Generation of iPSCs from Peripheral Blood Mononuclear Cells

PBMCs were collected from the blood of two healthy volunteers and two PD patients. All of the subjects gave their informed consent for inclusion before they participated in the study. The Jagiellonian University Bioethical Committee in Kraków approved the study by decision number KBET/173/B/2012 from 31 May 2012 and its prolongation from 17 December 2015 and 23 June 2016. PBMCs were isolated from whole blood on Pancoll (PAN Biotech, Aidenbach, Germany) gradient and cultured in Expansion Medium for 7 days. The medium was changed every other day. At day 0 PBMCs were transduced with Sendai Viral Vector—based on CytoTune^®^ 2.0 Sendai (Thermo Fisher Scientific) reprogramming vectors. PBMCs were counted and plated at density of 2.5 × 10⁴ cells per well of 96-well plate. The cells were infected by three vectors encoding different factors: KOS at Multiplicity of Infection, MOI = 5, hc-Myc at MOI = 5, hKlf4 at MOI = 3. After 24 h, the cells were collected and then centrifuged to wash out the vectors. After the next 24 h, the cells were plated on iMEF-cells from one well were transferred to a 100 mm dish. At day 5 and 7, the medium was changed to mixture of MEF medium and iPSCs medium in proportion 1:1. From day 9, the medium was changed every 2–3 days for fresh iPSCs medium. From day 21 to 28, the colonies were picked and transferred—one colony per well of 12-well plate coated with iMEF. Several iPSCs clones that were derived from two healthy volunteers and two PD patients were characterized for pluripotency. One representative clone from healthy volunteer and PD patient was selected for further research involving differentiation because the results were similar. To take the variability of the results into account, around 500 embryoid bodies were generated per healthy volunteer and PD patient and from them 48 were used to generate organoids, as described below.

### 4.3. Reverse Transcription-Polymerase Chain Reaction

The gene expression was analyzed at mRNA level by RT-PCR. Total RNA was isolated by Universal RNA/miRNA Purification Kit (Eurx), according to the manufacturer’s instruction. After measurement of RNA concentration, reaction of reverse transcription (RT) was performed with M-MLV reverse transcription kit (Promega, Madison, WI, USA).

### 4.4. Polymerase Chain Reaction Analysis

For PCR reactions, Taq PCR Master Mix (Eurx) and primers with optimal annealing temperature of 55 °C were used. The sequences of primers (5′ to 3′): OCT4 for: ATGGCGGGACACCTGGCTT, Oct4 rev: GGGAGAGCCCAGAGTGGTGACG, NANOG for: TGAACCTCAGCTACAAACAG, NANOG rev: TGGTGGTAGGAAGAGTAAAG, TERT for: TGTGCACCAACATCTACAAG, TERT rev: GCGTTCTTGGCTTTCAGGAT, GAPDH for: CAAAGTTGTCATGGATGACC, GAPDH rev: CCATGGAGAAGGCTGGGG. Primers for Sendai virus genome (SeV) were provided by Sendai Virus Kit.

### 4.5. Quantitative Reverse Transcription Real Time Polymerase Chain Reaction

Gene expression was analyzed at mRNA level by qRT-PCR. The reactions were performed using Quant Studio 7 Flex System (Applied Biosystems, Foster City, CA, USA) with Blank qPCR Master Mix 2× (Eurx) and the TaqMan Expression Assays (NURR1 Hs01117527_g1, TH Hs00165941_m1, TUBB Hs00801390_s1, PTX3 Hs00173615_m1, LMX1A Hs00898455_m1, FOXA2 Hs00232764_m1) (Thermo Fisher Scientific). The mRNA expression level of different genes was normalized to the housekeeping gene GAPDH (Hs02758991_g1), using the 2^−ΔCt^ method.

### 4.6. Immunofluorescent Staining

The organoids were washed with DPBS and then fixed in 4% paraformaldehyde for 24 h at room temperature. After incubation, they were embedded into paraffin blocks and then 3 µm slides were prepared in the Pathology Laboratory of the Children Hospital in Cracow. The slides were rinsed with TBS containing 0.025% Triton X-100 (Sigma–Aldrich) twice for 5 min and they were then were blocked in 1% bovine serum albumin (BSA, Sigma–Aldrich) in tris-buffered saline (TBS, 50 mM Tris-Cl, pH 7.6; 150 mM NaCl) for 1 h at room temperature. Subsequently, the slides were incubated with appropriate primary antibody diluted in 1% BSA in TBS for 1 h at 4 °C. Antibodies were as follows: mouse anti-tubulin antibody, beta III isoform (Tuj1 MAB1637, Sigma–Aldrich), rabbit anti-tyrosine hydroxylase antibody (TH AB152, Merck-Millipore, CA, USA). The slides were washed with TBS containing 0.025% Triton X-100 twice for 5 min and were incubated with Hoechst (Sigma–Aldrich) and secondary goat anti-rabbit or anti-mouse antibodies that were conjugated with Alexa Fluor 555 (Thermo Fisher Scientific) or Alexa Fluor 488 (Thermo Fisher Scientific) diluted in 1% BSA in TBS for 1 h at room temperature in the dark.

### 4.7. Alkaline Phosphatase Staining

The cells were washed with PBS and then fixed in 4% paraformaldehyde for 10 min Subsequently, the cells were washed three times with PBS and staining solution (H_2_O, 1 M Tris-HCl pH 9.5, 5 M NaCl, 1 M MgCl2, NBT-BCIP (Roche) was added. The cells were incubated for 10 min in the dark at room temperature and then washed with PBS. The results were analyzed with IX70 microscope (Olympus Corporation, Tokyo, Japan).

### 4.8. Teratoma Formation Assay

iPSCs were suspended in growth factor reduced Matrigel (Corning) and PBS (1:1). Approximately 2 × 10^6^ cells were injected into the adult female non-obese diabetic/severe combined immunodeficiency (NOD-SCID) mouse into the left dorsal flank. Mice health was controlled every day and the formation of tumors was analyzed. The teratomas were excised approximately two months after injection. After teratomas removal, hematoxylin-eosin (H/E) staining was performed. The 2nd Local Institutional Animal Care and Use Committee (IACUC) in Krakow approved all of the experimental protocols by decision numbers 162/2015 and 11/2018.

### 4.9. Embryoid Bodies Formation

iPSCs at 80% of confluence were incubated in dispase (Thermo Fisher Scientific) for 5–10 min to dissociate colonies to cell clumps. The dissociated colonies were washed with DPBS and centrifuged. The aggregates of cells were suspended in iPSCs medium without bFGF. The embryoid bodies were formed for four days. The medium was replaced every day. Around 500 embryoid bodies were generated per healthy volunteer and PD patient.

### 4.10. Midbrain Organoids Formation

The formation of midbrain organoids was performed with modifications, as described before [15]. 48 embryoid bodies per healthy volunteer and PD patient were used to generate organoids and those experiments were repeated in two independent time points. In each time point, three independently and randomly selected organoids per healthy volunteer and PD patient were characterized. Briefly, after four days, the single embryoid body was transferred to one well of V-shaped 96 well plate and cultured in neuronal induction medium DMEM F12/Neurobasal 1:1 (Thermo Fisher Scientific) that was supplemented with N2 1:100 (Thermo Fisher Scientific), B27–vit A 1:50 (Thermo Fisher Scientific), aminoacids 100 µM (Thermo Fisher Scientific), B-mercaptoethanol 0.1% (Sigma–Aldrich), heparin 1 µg/mL (Sigma–Aldrich), SB 431542 10 µM (Sigma–Aldrich), Noggin 200 ng/mL (PeproTech), CHIR 99021 0.8 µM (Sigma–Aldrich), Penicillin/Streptomycin 100 U/mL (Thermo Fisher Scientific) for four days. Medium was changed every day. Subsequently, the organoids were cultured in medium with midbrain patterning factors: FGF8 100 ng/mL (PeproTech) and SHH-C25II 100 ng/mL (PeproTech) for three days. Next, when neuroectodermal buds were observed, the medium was aspirated, and 30 µl of Matrigel was added to every well. After 30 min. of incubation in 37 °C, tissue growth induction medium containing Neurobasal (Thermo Fisher Scientific) supplemented with N2 1:100 (Thermo Fisher Scientific), B27–vit A 1:50 (Thermo Fisher Scientific), aminoacids 100 µM (Thermo Fisher Scientific), B-mercaptoethanol 0.1% (Sigma–Aldrich), glutamax 1% (PAN Biotech), insulin 2.5 µg/mL (Sigma–Aldrich) laminin 200 ng/mL (Thermo Fisher Scientific) Penicillin/Streptomycin 100 U/mL (Thermo Fisher Scientific), SHH-C25II 100 ng/mL (PeproTech), and FGF-8 100 ng/mL (PeproTech). After 24-h of incubation in Matrigel and tissue growth induction medium, the organoids were transferred into non-adherent 24 well plate (Sarstedt, Nümbrecht, Germany) and they were cultured in final organoid differentiation medium while using an orbital shaker. The final medium (Neurobasal) was supplemented with BDNF 10 ng/mL (PeproTech), GDNF 10 ng/mL (PeproTech), ascorbic acid 100 µM (Sigma–Aldrich), db-cAMP 125 µM (Sigma–Aldrich), and was changed every three days.

### 4.11. Statistical Analysis

Statistical analysis of the data was performed with GraphPad Prism Version 8.2.1. Student t-test was performed to verify statistically relevant differences between compared groups in each time point. Three independent organoids were analyzed per group. Data are presented as mean ± standard deviation. * represents statistically significant differences between groups (*p* < 0.05).

## 5. Conclusions

Our results suggest that organoids formation is powerful tool for in vitro modeling of PD disease. Our novel model identified several crucial gene candidates that might be responsible for development of idiopathic form of PD, such as TH, PTX3, LMX1A and FOXA2.

## Abbreviations

PD	Parkinson’s Disease
iPSCs	Induced Pluripotent Stem Cells
PBMCs	Peripheral Blood Mononuclear Cells
ESCs	Embryonic Stem Cells
EBs	Embryoid Bodies
mDA	Midbrain Dopaminergic neurons
MEFs	Mouse Embryonic Fibroblasts
SeV	Sendai viral Vector
